# Survival-Weighted Health Profiles in Nasopharyngeal Cancer Patients

**DOI:** 10.3389/fonc.2021.635667

**Published:** 2021-03-15

**Authors:** Chia-Hsuan Lai, Wen-Cheng Chen, Chiung-Cheng Fang, Miao-Fen Chen

**Affiliations:** ^1^ Department of Radiation Oncology, Chang Gung Memorial Hospital, Chiayi, Taiwan; ^2^ School of Medicine, College of Medicine, Chang Gung University, Taoyuan, Taiwan; ^3^ Graduate Institute of Clinical Medicine, College of Medicine, Chang Gung University, Taoyuan, Taiwan

**Keywords:** quality of life, quality-adjusted life expectancy (QALE), life expectancy, nasopharyngeal cancer, survival-weighted psychometric scores

## Abstract

**Introduction:**

In treating nasopharyngeal cancer (NPC) patients, excellent tumor control and patient survival rates can be achieved in the era of intensity-modulated radiotherapy (IMRT). However, treatment-related toxicities affect the quality of life (QoL) of NPC survivors. This study was devised to estimate the life expectancy (LE), quality-adjusted life expectancy (QALE) and survival-weighted psychometric scores (SWPS) in NPC patients.

**Methods:**

A sample of 875 non-metastatic NPC patients diagnosed between January 1, 2009 and June 30, 2013 was collected for estimation of lifetime survival function. All patients were followed up until death or censored on December 31, 2015. To obtain the utility and psychometric score for estimation of LE, QALE, and SWPS, 99 patients were measured with the Taiwanese version of the EuroQol instrument (EQ-5D) and the Taiwan Chinese versions of the European Organization for Research and Treatment of Cancer (EORTC) Quality of Life Questionnaire (QLQ)-C30 and QLQ-H&N35 between October 1, 2013 and December 31, 2017. By utilizing linear extrapolation of a logit-transformed curve, the LE of NPC patients can be estimated. The QALE and SWPS can be obtained by combining the LE and the corresponding QOL function.

**Results:**

The mean age of the 875 non-metastatic NPC patients was 50.3 years. The estimated average LE and QALE for NPC patients and for the reference population were 15.5 years and 14.3 quality-adjusted life years (QALYs) and 29.5 years and 29.5 QALYs, respectively. On average, the estimated lifelong duration of pain and painkiller use were 6.0 years and 2.2 years. The estimated lifelong duration of impairment of swallowing, speech, smell and taste were 14.0, 9.8, 8.7, and 7.5 years, respectively. The estimated lifelong duration of problems with dry mouth, teeth, emotion, fatigue, sleep, and social contact were 13.4, 10.1, 9.1, 12.3, 6.7, and 4.5 years, respectively. The estimated lifelong duration of tube-feeding was 1.3 months.

**Conclusions:**

The estimated LE and QALE for NPC patients were 15.5 years and 14.3 QALYs. Furthermore, SWPS could help people understand more about the impact of radiotherapy on NPC patients. These data could also be useful for policy makers to allocate limited resources in health care.

## Introduction

In treating nasopharyngeal cancer (NPC) patients, excellent tumor control and patient survival rates were shown in the era of intensity-modulated radiotherapy (IMRT) (1–4). Although a majority of patients can be cured and become long-term survivors, radiation-related toxicities usually affect the quality of life (QoL) of NPC survivors (5, 6). In general, comprehensive outcome assessments for NPC patients should include survival and QoL in estimating quality-adjusted life expectancy (QALE) and survival-weighted psychometric scores (SWPS). Combining the survival function with a mean QoL at different time points, these goals can be carried out (7–9). This study was devised to estimate the life expectancy (LE), QALE, and SWPS in NPC patients treated by IMRT.

## Materials and Methods

### Patients

**Figure 1:**
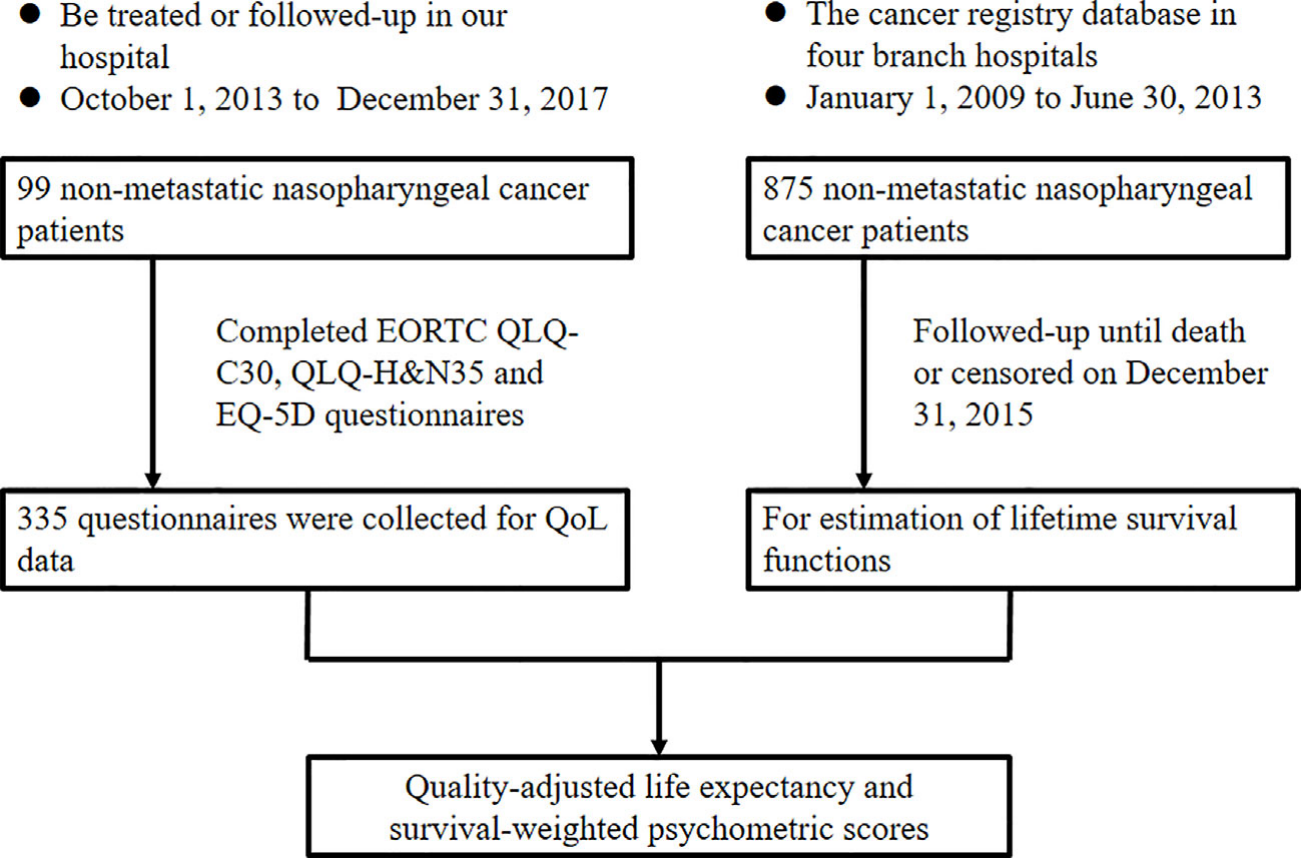
Flowchart of the study design. EORTC, European Organization for Research and Treatment of Cancer; QLQ, quality of life questionnaire; H&N, head and neck; EQ-5D, EuroQol- 5 Dimension; QoL, quality of life.

To obtain the QoL data, NPC patients were enrolled in this study if they were treated or followed-up at our institution between October 1, 2013 and December 31, 2017. Other eligibility criteria included age over 18 years old, an Eastern Cooperative Oncology Group performance status of 0–2, no distant metastasis at diagnosis, and no other primary cancer. All patients who completed questionnaires signed a written informed consent before study enrollment. This study was approved by our institutional review board. **Figure 1** showed the flow chart of the study design.

Pretreatment workup included history and physical examination, flexible nasopharyngoscopy, computed tomography, or magnetic resonance imaging of the head and neck, chest X-ray, bone scan, and abdominal ultrasound. The 2010 American Joint Committee on Cancer (AJCC) staging system was used for disease staging. All patients were treated by IMRT with or without chemotherapy.

A cohort of 875 non-metastatic NPC patients diagnosed between January 1, 2009, and June 30, 2013, was collected from the cancer registry database in four branch hospitals of our hospital system for estimation of lifetime survival functions. All 875 patients were followed-up until death or censored on December 31, 2015.

### QOL Instruments

Taiwan Chinese versions of the questionnaires of the European Organization for Research and Treatment of Cancer (EORTC), Quality of Life Questionnaire (QLQ)-C30, and QLQ-H&N35 were acquired from the Quality of Life Unit, EORTC Data Center in Brussels, Belgium (10–12). The multiple-item and single-item scales in the EORTC QLQ-C30 and QLQ-H&N35 ranged in score from 0–100. A higher score in functioning/QoL scales indicated a better level of functioning/QoL. A higher score in symptoms/problems scales represented a worse level of symptoms/problems.

The Taiwanese version of the EuroQol instrument (EQ-5D) was developed and validated by Chang et al. (13). The EQ-5D (14) is a tool for measuring generic health status, which is commonly used in cost-utility analysis. It comprises five functional domains: mobility, self-care, usual activities, pain/discomfort, and anxiety/depression. Each domain has three levels: no problems, some/moderate problems, and severe/extreme problems. The health state description from the five domains was converted into one utility value by the time trade-off method in Taiwan (15). The utility value lies in the interval from zero to one, in which one indicates full health.

### Statistical Analysis

The survival time was measured from the initiation of radiotherapy. Patients were followed until death or the end of the study on December 31, 2017. Overall survival was determined by the Kaplan-Meier method. All statistical analyses were performed using SPSS software for Windows, version 17.0 (SPSS, Chicago, IL, USA).

Using the life table of the general population in Taiwan, the survival function of the reference population was determined by the Monte Carlo method. To acquire the LE of NPC patients (an extrapolation of 50 years), linear extrapolation of a logit-transformed curve of the survival ratio between NPC patients and the reference population was employed. All the details were represented in former studies (7–9). By utilizing kernel-smoothing the cross-sectional QoL data from a random sample of patients, the estimated average QoL function was obtained (7). For every time interval from the beginning of radiotherapy to the date of QoL data obtained, the survival outcome of the cohort was multiplied by the utility value or psychometric scores of every patient to estimate the QALE or the SWPS. The utility value for the living reference population was assumed to be 1. The minimal requirement of the sample size was 50 to create the mean QoL function curve with time based on the previous study (7). The iSQoL software (http://sites.stat.sinica.edu.tw/isqol/) was used for the extrapolation. In previous studies, this method has been validated (16–20).

In this study, the LE, QALE, and SWPS of NPC patients were estimated from follow-up data over a 7 year period with an extrapolation of 50 years.

## Results

### Patient Characteristics

A cohort of 875 non-metastatic NPC patients diagnosed between January 1, 2009 and June 30, 2013 was collected for estimation of lifetime survival functions. **Table 1** shows the patient characteristics. The clinical stage distributions were as follows: stage I (n = 79; 9%), stage II (n = 182; 20.8%), stage III (n = 277; 31.7%), and stage IV (n = 337; 38.5%). Seven hundred and ninety-two patients (90.5%) received combination chemotherapy.

**Table 1:** Patient characteristics.

Variables	All patients (N=875)	Patients with QoL questionnaires (N=99)	*p value
Mean age at diagnosis, years (± SD)	50.3 (± 12.6)	47.5( ± 11.4)	^&^0.03
Gender			^#^0.97
Male	688 (78.6%)	78 (78.8%)	
Female	187 (21.4%)	21 (21.2%)	
AJCC stage, 7^th^ ed.			^#^0.12
I	79 (9.0%)	14 (14.1%)	
II	182(20.8%)	17 (17.2%)	
III	277 (31.7%)	38 (38.4%)	
IV	337 (38.5%)	30 (30.3%)	
T stage			^#^<0.01
T1	378 (43.2%)	42 (42.4%)	
T2	85 (9.7%)	23 (23.2%)	
T3	191 (21.8%)	19 (19.2%)	
T4	221 (25.3%)	15 (15.2%)	
N stage			^#^0.11
N0	137 (15.6%)	18 (18.2%)	
N1	320 (36.6%)	26 (26.3%)	
N2	258 (29.5%)	39 (39.4%)	
N3	160 (18.3%)	16 (16.1%)	
Histology			^$^0.50
WHO type 1	17 (1.9%)	2 (2.0%)	
WHO type 2	156 (17.8%)	13 (13.1%)	
WHO type 3	702 (80.2%)	84 (84.9%)	
Combination with chemotherapy			^#^0.40
No	83 (9.5%)	14 (14.1%)	
Yes	792 (90.5%)	85 (85.9%)	

^&^Independent sample t tests; ^#^Chi-square test; ^$^Fisher’s exact test.

QoL, quality of life; SD, standard deviation; RT, radiotherapy; AJCC, The American Joint Committee on Cancer; WHO, World Health Organization.

During the study period, 99 patients were enrolled and were measured with questionnaires of the Taiwanese version of the EQ-5D and the Taiwan Chinese versions of the EORTC QLQ-C30 and QLQ-H&N35. A total of 335 questionnaires were collected. **Table 2** showed the mean scores of the EORTC QoL scales.

**Table 2:** The mean scores of the EORTC QoL scales.

	Scores (± SD)
EORTC QLQ-30	
Global quality of life	60 (± 22)
Physical functioning	88 (± 16)
Emotional functioning	83 (± 20)
Cognitive functioning	82 (± 19)
Social functioning	78 (± 25)
Role functioning	88 (± 24)
Fatigue	30 (± 23)
Nausea/vomiting	10 (± 19)
Pain	20 (± 26)
Dyspnea	10 (± 19)
Insomnia	22 (± 28)
Appetite loss	26 (± 31)
Constipation	17 (± 22)
Diarrhea	9 (± 16)
Financial problems	26 (± 32)
EORTC QLQ-H&N35	
Pain	16 (± 20)
Swallowing	24 (± 22)
Senses (taste/smell)	32 (± 28)
Speech	16 (± 20)
Social eating	25 (± 26)
Social contact	9 (± 16)
Sexuality	23 (± 29)
Teeth	29 (± 31)
Opening mouth	18 (± 23)
Dry mouth	53 (± 34)
Sticky saliva	40 (± 34)
Coughing	26 (± 23)
Feeling ill	29 (± 29)

EORTC, European Organization for Research and Treatment of Cancer; QoL, quality of life; QLQ, quality of life questionnaire; H&N, head and neck; SD, standard deviation.

### The Survival Outcome, LE, and QALE

The median follow-up time was 50.4 months (range 1.9–84.9 months) for the 875 non-metastatic NPC patients. The 5 year overall survival was 77.7%. The estimated LE and QALE for NPC patients and for the reference population were 15.5 years and 14.3 quality-adjusted life years (QALYs) and 29.5 years and 29.5 QALYs, respectively.

### Symptoms or Functional Disabilities

**Figure 2:**
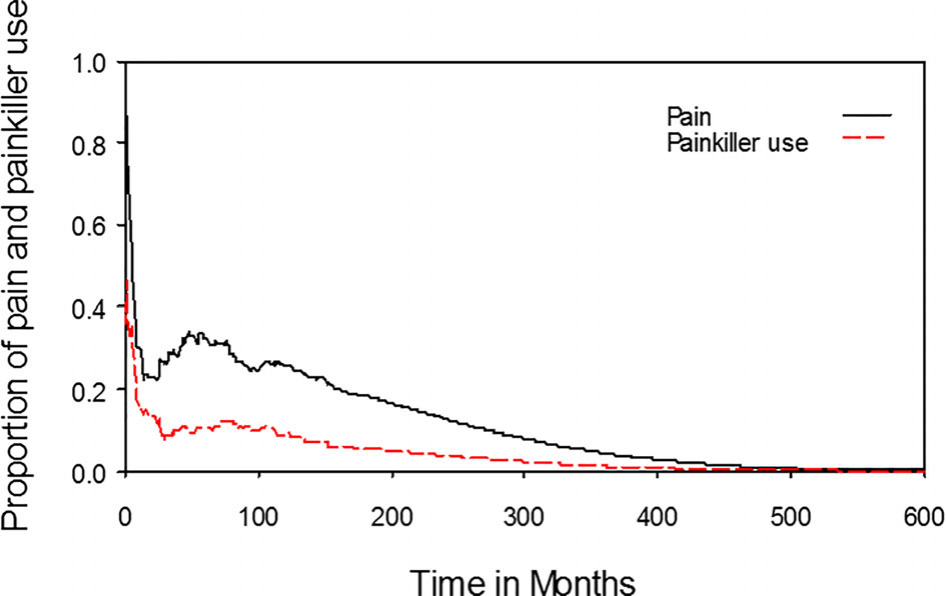
Dynamic change in pain and painkiller use for nasopharyngeal cancer patients.

**Figure 3:**
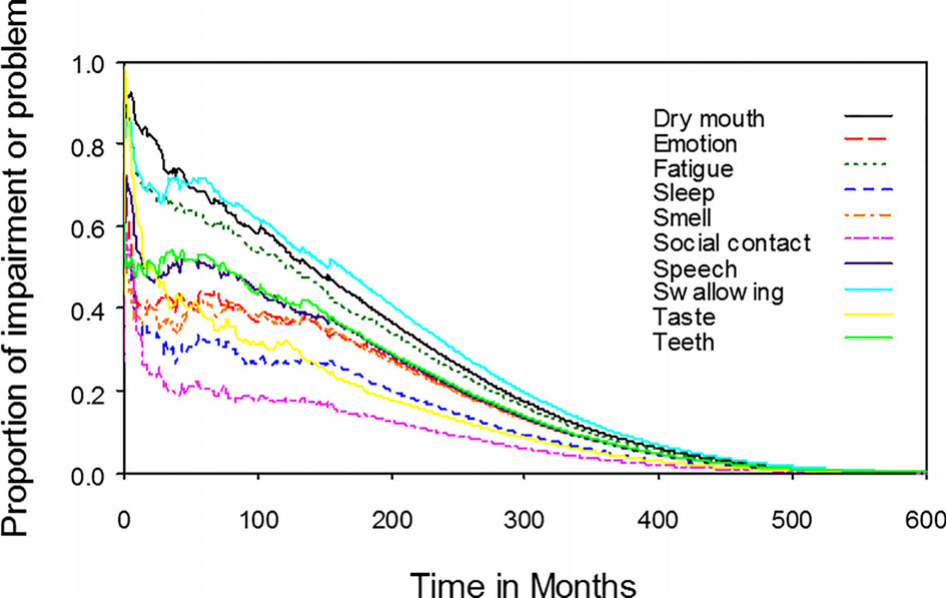
Dynamic change in different functional impairments or problems for nasopharyngeal cancer patients. The estimated lifelong duration of any functional impairments or problems were the areas under the quality-adjusted survival curves. The estimated lifelong duration of any impairment of swallowing, speech, smell, and taste were 14.0, 9.8, 8.7, and 7.5 years, respectively. The estimated lifelong duration of problems with dry mouth, teeth, emotion, fatigue, sleep and social contact were 13.4, 10.1, 9.1, 12.3, 6.7, and 4.5 years, respectively.

The median time from the start of radiotherapy to the date of completing questionnaires was 3.8 months (range, 0–179.5 months). The proportions of symptoms or functional disabilities among NPC patients receiving radiotherapy with an individual item (e.g., pain, swallowing, smell, and taste) scoring more than zero, which was equivalent to any symptom or disability, were plotted against time after the beginning of radiotherapy. On average, the estimated lifelong durations of pain and painkiller use were 6.0 and 2.2 years, respectively (**Figure 2**). The estimated lifelong duration of any impairment of swallowing, speech, smell, and taste were 14.0, 9.8, 8.7, and 7.5 years, respectively (**Figure 3**). The estimated lifelong duration of problems with dry mouth, teeth, emotional functioning, fatigue, sleep, and social contact were 13.4, 10.1, 9.1, 12.3, 6.7, and 4.5 years, respectively (**Figure 3**). The estimated duration of tube feeding was 1.3 months.

### Extrapolation Validity

**Figure 4:**
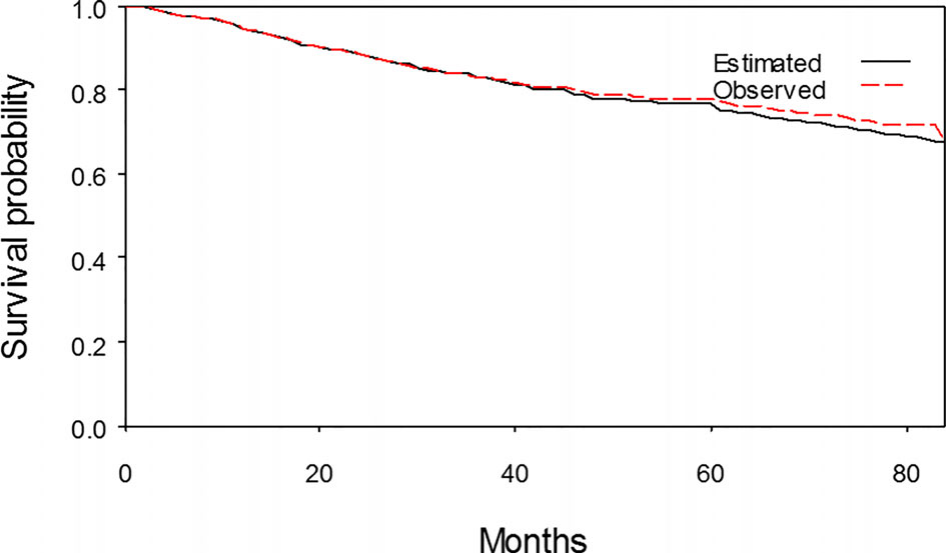
The estimated 7-year survival curve and the observed 7-year survival curve matched sufficiently.

The initial 5-year follow-up data (January 1, 2009–December 31, 2013) of the 875 NPC patients was used to predict the survival outcome for 2 years beyond December 31, 2013. An estimated 7-year overall survival rate was compared with the actual survival outcome obtained by the Kaplan-Meier method from January 1, 2009 to December 31, 2015. At the end of the 7-year follow-up, the estimated mean survival time (± SE) of the 875 NPC patients was 69.2 (± 0.6) months. There was 1.0% relative bias from the actual value (69.9 ± 0.9 months). The actual survival curve matched sufficiently with the estimated curve (**Figure 4**).

## Discussion

To the best of our knowledge, this was the first study to describe lifelong symptoms or functional disabilities in NPC patients. Patients had problems with swallowing, dry mouth, and fatigue for lifelong durations of 14.0 years, 13.4 years, and 12.3 years, respectively. In addition, patients had symptoms of pain for a lifelong duration of 6.0 years, but only 2.2 years of painkiller use. These data facilitated a more accurate estimation of NPC patients’ QoL after radiotherapy with or without chemotherapy. Furthermore, these data could also be useful for policy makers to allocate limited resources in health care.

Three studies addressed the QALE of NPC patients (17, 19–21). In our previous study (19), the QALE was estimated to 11.6 QALYs and the loss of QALE was 12.8 QALYs in NPC patients receiving IMRT with or without chemotherapy. However, the study used the score of global quality of life from the EORTC QLQ-C30 rather than the EQ-5D as the utility index. This might have underestimated the utility and the QALE (21). Hung et al. reported that the loss of QALE was 13.8 QALYs in male patients and 11.5 QALYs in female patients (20). The study used data from the Taiwan Cancer Registry from 1998 to 2009, but without consideration of the different techniques of radiotherapy during this period. Liao et al. reported the estimated LE and QALE of NPC patients were 15.5 years and 14.2 QALYs, respectively (21). The loss of LE and QALE were 15.8 years and 17.1 QALYs, respectively. Our present study showed similar results. The estimated average LE and QALE were 15.5 years and 14.3 QALYs, respectively. The estimated loss of LE and QALE were 14.1 years and 15.2 QALYs, respectively.

There were several limitations in this study. The QALE might have been overestimated because of an assumption of continuance of the same level of QoL as that near the end of follow-up during extrapolation. In general, the real QoL often declines with age (22, 23). Moreover, patients who completed the questionnaires might have had a better QoL than those confined at home. Those patients with a worse QoL were probably missed in the sampling. Furthermore, patients who survived longer might tend to have had a better QoL and vice versa (22). As a result, the QALE might have been overestimated due to the positive selection bias of the QoL outcomes. On the other hand, the utilities for the reference population were assumed to be 1 throughout the survival period. This might overestimate the QALE of reference population and also the estimated loss of QALE for NPC patients. In addition, the patients completed the questionnaires were younger than the cohort used for estimation of life expectancy. It might imply the QoL is better in patients completed the questionnaires and may result in the overestimation the QALE and the SWPS in functional scales. It might also cause the underestimation of the SWPS in symptom scales. However, the lifetime extrapolation was based on present and previous experiences (17). This method could underrate the actual survival of future cancer patients with the evolution and introduction of new skills in cancer treatment (20). Finally, only 99 patients completed the questionnaires, so further stratified analysis by potentially important factors was not feasible.

In summary, this study showed that the estimated LE and QALE of NPC patients treated with IMRT with or without chemotherapy were 15.5 years and 14.3 QALYs. Furthermore, SWPS, such as the lifelong duration of impairment of swallowing, speech, smell, and taste, and the lifelong duration of problems with pain, painkiller use, dry mouth, teeth, emotion, fatigue, sleep, and social contact could help people understand more about the impact of radiotherapy on NPC patients. These data could also be useful for policy makers to allocate limited resources in health care.

## Data Availability Statement

The raw data supporting the conclusions of this article will be made available by the authors, without undue reservation.

## Ethics Statement

The study was reviewed and approved by Chang Gung Medical Foundation Institutional Review Board (No.102-2668B). The patients/participants provided their written informed consent to participate in this study.

## Author Contributions

C-HL and W-CC were involved in the conception and design. C-HL, W-CC, C-CF, and M-FC were involved in the analysis and interpretation of the data. C-HL drafted the paper. W-CC, C-CF, and M-FC revised it critically for intellectual content. All authors contributed to the article and approved the submitted version.

## Funding

This study was supported by the Chang Gung Memorial Hospital (grant numbers: CORPG6D0261-3). The funding source had no role in the design of this study and had no role during its execution, analyses, interpretation of the data, or decision to submit results. 

## Conflict of Interest

The authors declare that the research was conducted in the absence of any commercial or financial relationships that could be construed as a potential conflict of interest.
